# Mental Illness in the Bible (Old and New Testament)

**DOI:** 10.1192/j.eurpsy.2025.1863

**Published:** 2025-08-26

**Authors:** N. Ramalho, T. Rocha, J. F. Cunha, J. C. Moura, J. Leal, D. Seabra, I. Lopes, G. Santos, M. Rosa

**Affiliations:** 1 Psychiatric and Mental Health Department, ULSAR, Barreiro, Portugal

## Abstract

**Introduction:**

The Bible offers various insights into human struggles, including what can be interpreted today as mental illness. Although ancient texts do not explicitly refer to mental health using modern terminology, there are many accounts of emotional distress, depression, anxiety, and other psychological challenges. Throughout Scripture, several figures are portrayed grappling with deep sorrow, fear, and mental turmoil. These narratives provide spiritual reflections on suffering, healing, and divine intervention, shedding light on how biblical teachings have historically addressed human fragility.

**Objectives:**

To raises questions about how we can relate ancient wisdom to contemporary mental health issues, offering opportunities for spiritual growth, empathy, and care in our own lives.

**Methods:**

A non-systematic review of the published literature was conducted using the Google Scholar database with the search terms “Bible” and “mental illness.” Articles were selected based on their relevance, and further information was obtained through direct consultation of biblical texts.

**Results:**

The prophet Elijah exhibits signs of reactive depression, triggered by stress after confronting the prophets of Baal and receiving a death threat from Jezebel (1 Kings 18:20-40). His symptoms—loss of appetite, isolation, low self-esteem, and hopelessness—are well-documented (1 Kings 19:3). God’s response (1 Kings 19:11-14) provides an example of care for depression, with affection, understanding, and patience.

James 5:15-18 references Elijah to highlight that depression can affect Christians, suggesting that illness, whether physical or spiritual, requires dialogue and support. James emphasizes God’s forgiveness, even if illness stems from sin, viewing depression as an organic condition in line with the holistic Jewish understanding. He advocates for confession and prayer as therapeutic (James 5:16), stressing that mercy triumphs over judgment.

Psalm 6:6-7 captures the deep despair of depression, showing the importance of seeking God amid mental anguish, which is often invisible to others.

**Conclusions:**

The accounts of figures like Elijah and the reflections in Psalms demonstrate that conditions resembling modern definitions of depression and anxiety have long been acknowledged, albeit through the lens of ancient cultural and religious contexts. The compassionate care that God extends to Elijah, coupled with the guidance found in the New Testament, particularly in the book of James, underscores the importance of community and support in addressing mental health challenges.

By examining these stories, we gain a broader understanding of how faith communities have interpreted and coped with the complexities of mental illness - in light of their relationship with God.

These accounts present a holistic biblical view of depression, underscoring the need for empathy, spiritual care, and community support in mental health.

**Disclosure of Interest:**

None Declared

